# Patient-reported outcome measures for anticipatory grief: a systematic review

**DOI:** 10.3389/fpsyg.2026.1709822

**Published:** 2026-02-11

**Authors:** Shiyuan Wang, Wangyang Gu, Ling Zhang, Wei Liu, Zeqin Tao, Yanrong Cheng, Wenlin Liu, Xiaoting Zhao, Wen Tu, Xing Gao

**Affiliations:** 1 School of Nursing, Hainan Medical University, Haikou, China; 2 School of Public Health, Hainan Medical University, Haikou, China; 3 College of Nursing, University of Utah, Salt Lake City, UT, United States

**Keywords:** anticipatory grief, chronic illness, patient-reported outcome measure, psychometric properties, systematic review

## Abstract

**Background:**

Anticipatory grief (AG) refers to the experience of grief symptoms by patients or their caregivers in response to life-threatening illnesses, even before an actual loss has occurred. The selection of valid and reliable patient-reported outcome measures (PROMs) to assess AG is essential for early identification, effective intervention, and the reduction of prolonged grief disorder. This study summarizes psychometric properties of AG PROMs and recommends the most effective PROMs.

**Methods:**

Nine databases (PubMed, EMBASE, Web of Science, CINAHL, Cochrane Library, PsycINFO, CNKI, Wanfang, China Biology Medicine Database) were searched from inception to December 2024. Search terms include “preparatory” or “preparedness” or “pre-loss” or “pre-death” or “anticipatory,” “grief” or “mourn” or “bereave,” “surveys and questionnaires” or “assess” or “instrument” or “measure” or “inventory” or “scale” or “interview.” Eligible studies included those reporting the development or validation of any AG assessment for patients with chronic illnesses and their informal caregivers. Extracted psychometric properties encompassed content validity, structural validity, internal consistency, cross-cultural validity, reliability, measurement error, criterion validity, hypothesis testing, and responsiveness. We conducted a reliability generalization meta-analysis of internal consistency (Cronbach’s alpha) for identical AG PROMs across different studies. Quality assessment, measurement property ratings, synthesis, and modified grading of evidence were conducted following the COSMIN methodology for systematic reviews.

**Results:**

A total of 20 studies comprising of 13 AG PROMs were included. 2 PROMs were designed for patient assessment, and 11 targeted informal caregivers. PG-12 demonstrated high-quality evidence in assessing informal caregiver AG, with satisfactory content validity and internal consistency, supporting grade A. MM-CGI-CCPS showed insufficient evidence for structural validity and internal consistency, warranting grade C; the remaining tools are suggested as grade B due to limited evidentiary support.

**Conclusion:**

This review confirms PG-12 as a grade A PROM for measuring AG in informal caregivers. However, no grade A PROMs were identified for assessing AG in patients. Future studies are needed to further validate the methodological quality and measurement properties of PROMs used in patient populations. Significant concerns remain regarding other existing PROMs, particularly in content validity, structural validity, cross-cultural adaptation, measurement error, and reliability, which increase uncertainty in available evidence.

**Systematic review registration:**

https://www.crd.york.ac.uk/PROSPERO/view/CRD42024624205, identifier (CRD42024624205).

## 1 Introduction

Chronic illnesses (CIs) have become the most significant global burden of disease. According to the World Health Statistics Report released by the World Health Organization (WHO) in 2024, about 43 million people died from CIs worldwide in 2021. These account for about three-quarters of all global deaths (GBD 2021 Forecasting Collaborators, 2024). According to the Global Report on CIs 2023 released by the WHO, CIs cost the world $52 trillion annually, accounting for 4.3% of the global average annual gross domestic product (GDP) (World Health Organization, 2023). Beyond the physical and economic strain, CIs also bring profound emotional challenges, as patients and their informal caregivers often begin grieving long before the end of life.

Anticipatory grief (AG) is a prevalent yet often overlooked psychological response characterized by the experience of grief symptoms before the actual occurrence of loss (Cheung et al., 2018). This phenomenon is particularly common among patients with CIs such as cancer, Alzheimer’s disease, and stroke, as well as their informal caregivers (Holm et al., 2019; Pérez-González et al., 2023; Liu et al., 2023). Research reveals that AG manifests not merely as cognitive anticipation of loss, but more significantly as a psychological burden that individuals continually bear (Chen et al., 2024).

AG has become a prevalent clinical phenomenon among patients and informal caregivers. Studies have shown that about 30–50% of patients with CIs would experience a psychological response to AG because of fear and uncertainty about the impending loss, which is particularly pronounced in cancer patients (Treml et al., 2021). If AG is not promptly identified and addressed, it can significantly diminish patients’ quality of life and negatively impact treatment adherence (Li et al., 2020). Moreover, studies have shown that elevated levels of AG in cancer patients are significantly associated with severe depression and anxiety, which can negatively impact health outcomes, reduce survival rates, and increase the risk of adverse events (Tian et al., 2021; Zheng et al., 2025).

Regarding patient care, about 14.9–33% of informal caregivers also experience AG due to excessive physical and mental burden, and uncertainty about the progress of the patients’ disease (Nielsen et al., 2017). This phenomenon occurs in primary informal caregivers, including spouses and parents. AG exacerbates with increasing caregiving stress, subsequently inducing psychological disorders such as anxiety and depression in informal caregivers, and may potentially lead to adverse bereavement outcomes (Areia et al., 2019). Previous studies have identified a significant correlation between AG in informal caregivers and poor patient prognosis, which not only compromises informal caregivers’ quality of life and caregiving capacity but also contributes to premature nursing institutionalization and increased early mortality among patients (Toot et al., 2017; Tekdemir et al., 2024). Early identification of AG can improve health outcomes and quality of life for both patients and their informal caregivers.

Despite growing recognition of AG among patients with CIs and their informal caregivers, there is currently no standardized, high-quality tool for its assessment. Existing evidence is limited, with only one systematic review focused on dementia informal caregivers (Dehpour and Koffman, 2023). As a result, researchers and clinicians lack reliable guidance when choosing appropriate instruments for different CIs populations, making it difficult to identify those at greatest risk for AG. The use of PROMs with good psychometric properties is essential for early identification and intervention for AG. This study aimed to systematically review and compare existing AG assessment tools, assessing their psychometric properties to provide evidence-based recommendations for selecting high-quality PROMs. The findings will inform the selection of the most appropriate assessment tools for clinical practice by enhancing the accuracy of AG identification in patients and informal caregivers, supporting timely and individualized interventions, and informing future guidelines to improve the well-being of patients with CIs and their informal caregivers.

## 2 Methods

### 2.1 Design

This study employed the Consensus-based Standards for the Selection of Health Measurement Instruments (COSMIN) method to systematically review the psychometric properties. We registered the current review in the PROSPERO database (registration number: CRD42024624205).

### 2.2 Search strategy

In the search strategy, three steps were followed. First, search terms were identified and standardized using authoritative thesauri. English terms were developed based on PubMed’s MeSH and relevant keywords, while Chinese terms were drawn from their official equivalent, the SinoMed Subject Headings system, to ensure the Chinese search terms are semantically equivalent to the English ones. The research team, including an academic librarian, a statistical analysis expert, and six researchers, were trained in evidence-based methodologies, confirmed the final search strategy, and both English and Chinese search terms included: “preparatory” or “preparedness” or “pre-loss” or “pre-death” or “anticipatory,” “grief” or “mourn” or “bereave,” “surveys and questionnaires” or “assess” or “instrument” or “measure” or “questionnaire” or “inventory” or “scale” or “interview” or “criteria.” Secondly, nine databases (PubMed, EMBASE, Web of Science, Cochrane Library, PsycINFO, CINAHL, CNKI, Wanfang, and China Biology Medicine Database) were selected from their inception to December 10, 2024. The search filter developed by the Oxford University PROMs group in PubMed was utilized to further enhance search accuracy in accordance with the COSMIN guidelines. Thirdly, relevant core journals and books from the past five years, including *Oxford Textbook of Palliative Medicine*, *Practical Guidance on Palliative Care in Oncology*, and *Palliative Medicine*, were manually searched, and the references of included studies were traced to supplement any eligible literature not included. Supplementary file 1 shows the search strategies.

### 2.3 Selection criteria

Inclusion criteria: (1) including all patients with CIs and their informal caregivers, with all participants aged ≥ 18 years. (2) studies focused on developing or validating tools to measure AG, including but not limited to questionnaires, checklists, and scales. (3) assessment tools contain at least one psychometric feature for measuring AG. (4) studies published in English or Chinese.

Exclusion criteria: (1) the full text is unavailable. (2) duplicate or overlapping publications. (3) review articles. (4) studies where the assessment instruments were employed exclusively as validation criteria for other PROMs. (5) used PROMs as an outcome measure tool.

### 2.4 Quality appraisal

Quality appraisal consisted of two steps: First, the COSMIN guidelines provided a standardized framework to evaluate measurement tools, ensuring a structured and comprehensive assessment of methodological quality and psychometric properties to identify high-quality PROMs. Second, the modified Grading of Recommendations, Assessment, Development and Evaluation (GRADE) system was used to assess the certainty of evidence. Two researchers trained in evidence-based methods, independently conducted the appraisal. Discrepancies were resolved through consultation with a third researcher to reach consensus, ensuring reliability and accuracy.

#### 2.4.1 Methodological quality assessment

The methodological quality of each study was evaluated using the COSMIN risk bias checklist (Mokkink et al., 2018). The checklist comprises 10 domains and 116 items that assess: PROM development, content validity, structural validity, internal consistency, cross-cultural validity/measurement invariance, reliability, measurement error, criterion validity, hypothesis testing, and responsiveness. Each item was rated on a 4-point scale: “very good,” “adequate,” “doubtful,” and “inadequate.” The “not applicable (NA)” rating was assigned to items deemed irrelevant or absent in a given study and was excluded from the final domain evaluation. The overall rating for each domain was determined using the “worst-score-counts” principle, whereby the lowest item score defined the final domain evaluation.

#### 2.4.2 Summarizing the quality of psychometric properties

The measurement properties of PROMs were assessed according to the COSMIN standard (Terwee et al., 2007), covering nine domains (excluding PROM development). Each property was categorized into three levels: “sufficient (+),” “insufficient (−),” or “indeterminate (?).” For content validity, the following criteria were applied: “sufficient (+)” if ≥ 85% of items met the standards, “insufficient (−)” if not met, and “indeterminate (?)” when information was inadequate or when there was a high risk of bias. Items were rated as “not applicable (NA)” when they were irrelevant to the study content or when content was missing, and these items were excluded from the final evaluation. A consensus rating was established when individual study ratings were consistent; unresolved discrepancies in the assessments were labeled as ‘inconsistent’. Since COSMIN standards do not encompass exploratory factor analysis (EFA) for structural validity, we adopted the criteria proposed by Lee et al. (2019), rating this property as “sufficient (+)” when either ≥ 50% of the variance was explained or when Pearson’s correlation coefficient was ≥ 0.80, thereby ensuring a robust assessment of structural validity.

To evaluate the overall internal consistency of AG PROMs, reliability generalization (RG) meta-analyses were conducted separately for the Cronbach’s alpha (*α*) coefficients obtained from the total scale scores of each measurement instrument, with the pooled results classified as “sufficient (+)” or “insufficient (−).” To account for between-study heterogeneity, all analyses were based on random-effects models. Because Cronbach’s *α* is bounded between 0 and 1, its sampling distribution is non-normal; therefore, this study applied the Bonett transformation (Bonett, 2002) to correct for this issue. We performed the RG meta-analysis using inverse-variance weighting based on the sampling variances of the Bonett-transformed *α* values, and estimated between-study variance (*τ*^2^) using restricted maximum likelihood (López-López et al., 2013; Borenstein et al., 2009). For each meta-analysis, the pooled Cronbach’s *α* and its 95% confidence interval (CI) were computed using the Hartung–Knapp method (Hartung and Knapp, 2001). Heterogeneity was quantified using Cochran’s *Q* statistic and the 𝐼^2^ index. A statistically significant *Q* test (*p <* 0.05) was interpreted as evidence of heterogeneity (Higgins and Thompson, 2002). The magnitude of heterogeneity was interpreted based on 𝐼^2^ values as follows: negligible (*<* 25%), low (25–49.9%), moderate (50–74.9%), and high (*>* 75%). Forest plots were constructed to visually display study-specific *α* coefficients and the pooled estimates. This RG meta-analysis was reported in accordance with the REGEMA checklist (Sánchez-Meca et al., 2021).

#### 2.4.3 Grading of the quality of the evidence

We employed a modified GRADE approach to evaluate the quality of evidence for the psychometric properties of PROMs (Mokkink et al., 2016), including four criteria: risk of bias, inconsistency, indirectness, and imprecision. The evidence quality was then categorized into four levels: “high,” “moderate,” “low,” and “very low.”

Based on the evidence quality and grading, final recommendations were made for the included PROMs (Prinsen et al., 2018). The recommendation criteria were classified into three categories: grade A (strongly recommended): PROMs demonstrating sufficient (+) content validity (acceptable at any evidence level) and sufficient (+) internal consistency (supported by at least low-quality evidence); grade B (weakly recommended): PROMs showing potential for use but requiring further validation; grade C (not recommended): PROMs with high-quality evidence for a measurement property as insufficient (−).

### 2.5 Data extraction

Microsoft Excel 16.0 software was used to extract the basic information from each study and the characteristics of PROMs. Two researchers independently extracted the data, including basic information (author (year), PROMs, country/region, PROM language, research design, sample size and participants, participants’ mean age, Year of development/validity) and the characteristic information of PROMs (PROMs, references, Cronbach’s alpha, mode of administration, items number and Subscale, range of scores, original language).

### 2.6 Synthesis

References retrieved from relevant databases were imported into EndNote, and duplicates were removed. Two researchers trained in evidence-based methodologies independently screened the titles and abstracts of the retrieved articles. Articles meeting the inclusion criteria were subsequently reviewed in full text by the same two researchers. During both screening phases, any disagreements were resolved through discussion with a third researcher to reach consensus.

## 3 Results

### 3.1 Literature results

**Figure 1:**
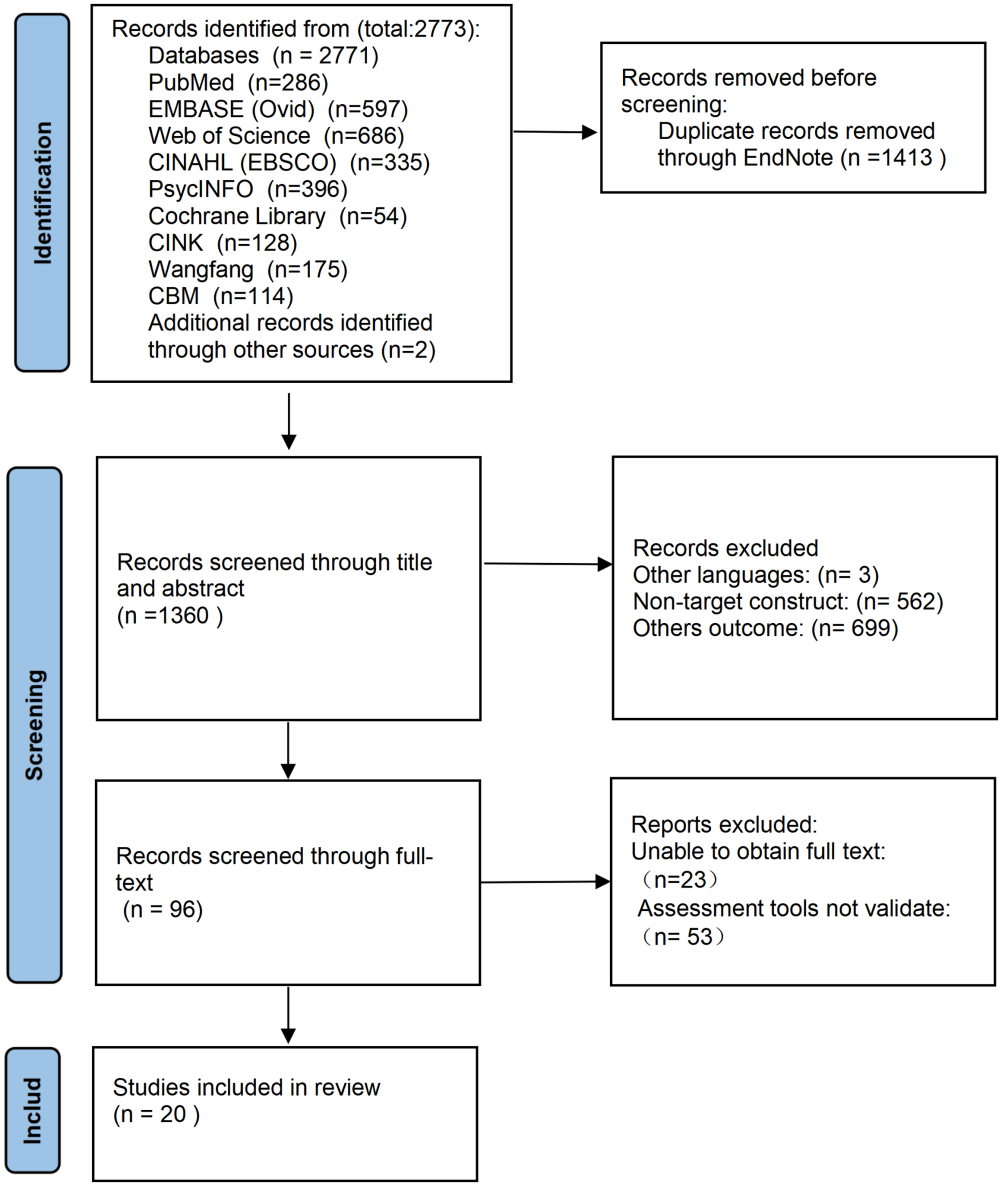
PRISMA flowchart of the identification and selection of studies.

A total of 2,773 articles were retrieved from a search of nine databases and a hand search of relevant core journals and books. After removing duplicates, a total of 1,413 articles remained (Figure 1). Following title/abstract and full-text screening, 1,360 articles were excluded because they did not meet the inclusion criteria or were review articles. After full-text screening, a total of 20 articles were included (Theut et al., 1991; Marwit and Meuser, 2002; Marwit and Meuser, 2005; Mystakidou et al., 2005; Periyakoil et al., 2005; Al-Gamal et al., 2009; Al-Gamal and Long, 2014; Liew, 2016; Meichsner et al., 2016; Chan et al., 2017; Coelho et al., 2017; Xin, 2017; Liew and Yap, 2018; Liew et al., 2018; Ar-Karci and Karanci, 2019; Cheng et al., 2019; Holm et al., 2019; Gilsenan et al., 2022; Önal et al., 2023; Liu et al., 2023). Supplementary file 2 contains the list of included studies.

### 3.2 Basic characteristics of the included studies

A total of 20 studies were included in the review, with 18 published in English and 2 in Chinese, as shown in Table 1. The articles were published between 1991 and 2024 and conducted across 11 regions: the United States, the United Kingdom, Greece, China, Hong Kong, Jordan, Singapore, Portugal, Turkey, Germany, and Sweden. All the studies were cross-sectional studies, with sample sizes ranging from 27 to 508 participants, involving a total of 4,978 participants. The subjects comprised patients (Mystakidou et al., 2005; Periyakoil et al., 2005; Xin, 2017) and informal caregivers (Theut et al., 1991; Marwit and Meuser, 2002; Marwit and Meuser, 2005; Al-Gamal et al., 2009; Al-Gamal and Long, 2014; Liew, 2016; Meichsner et al., 2016; Chan et al., 2017; Coelho et al., 2017; Xin, 2017; Liew and Yap, 2018; Liew et al., 2018; Ar-Karci and Karanci, 2019; Cheng et al., 2019; Holm et al., 2019; Gilsenan et al., 2022; Önal et al., 2023; Liu et al., 2023). The study participants included patients with CIs and their informal caregivers. The spectrum of chronic conditions encompassed dementia, cancer, stroke, and cerebral palsy. Two studies (Liew and Yap, 2018; Liew et al., 2018) used the same data to develop and validate separate scales. One article (Xin, 2017) includes the results of the measurement properties of AG measurement tools for two different groups of study subjects: patients and informal caregivers.

**Table 1:** Study characteristics.

Author (year)	PROMs	Country/region	PROMs language	Research design	Sample size and participants	Participants’ mean age	Year of development/validity
Theut et al. (1991)	AGS	USA	English	Cross-sectional design	27 caregivers of dementia patients	68.10	1991
Marwit and Meuser (2002)	MM-CGI	USA	English	Cross-sectional design	166 caregivers of dementia patients	Adult child:51.81 ± 8.05 Spouse: 71.47 ± 8.93	2002
Mystakidou et al. (2005)	PGAC	Greece	Greek	Cross-sectional design	451 advanced cancer patients	61.70	2003
Marwit and Meuser (2005)	MM-CGI-SF	USA	English	Cross-sectional design	292 caregivers of dementia patients	NR	2005
Periyakoil et al. (2005)	TIGDS	USA	English	Cross-sectional design	55 terminally ill patients	NR	2005
Al-Gamal et al. (2009)	MM-CGI-CCS	Jordan	English	Cross-sectional design	140 parents of children with cancer	Fathers: 39.9; Mothers: 34.4	2006
Al-Gamal and Long (2014)	MM-CGI-CCPS	Jordan	Arabic and English	Cross-sectional design	204 parents of children with cerebral palsy	34.70 ± 7.33	2010
Meichsner et al. (2016)	CGS	Germany	German	Cross-sectional design	229 caregivers of dementia patients	63.80 ± 10.50	2014
Liew (2016)	MM-CGI	Singapore	English	Cross-sectional design	72 caregivers of dementia patients	50.90 ± 11.60	2014
Xin (2017)	C-PGAC	China	Chinese	Cross-sectional design	388 avanced cancer patients	54.08 ± 12.10	2015
Xin (2017)	C-AGS	China	Chinese	Cross-sectional design	391 caregivers of cancer patients	NR	2015
Coelho et al. (2017)	PG-12	Portugal	Portuguese	Cross-sectional design	94 caregivers of cancer patients	52.02 ± 12.87	2016
Chan et al. (2017)	C-MM-CGI-SF	Hong Kong	Cantonese Chinese	Cross-sectional design	120 caregivers of dementia patients	55.46 ± 14.89	2017
Liew and Yap (2018)	MM-CGI-BF	Singapore	English	Cross-sectional design	394 caregivers of dementia patients	53.0 ± 10.70	2018
Liew et al. (2018)	C-MM-CGI	Singapore	Chinese	Cross-sectional design	394 caregivers of dementia patients	53.0 ± 10.70	2018
Ar-Karci and Karanci (2019)	T-MM-CGI-SF	Turkey	Turkish	Cross-sectional design	190 caregivers of dementia patients	51.41 ± 8.67	2018
Holm et al. (2019)	AGS-13	Sweden	Swedish	Cross-sectional design	270 caregivers of palliative care patients	61.0 ± 14.00	2014
Cheng et al. (2019)	CGQ	Hong Kong	Chinese	Cross-sectional design	173 caregivers of dementia caregivers	58.27 ± 10.42	2017
Gilsenan et al. (2022)	MM-CGI-SF	UK	English	Cross-sectional design	508 caregivers of dementia patients	54.00 ± 12.90	2020
Önal et al. (2023)	T-PG-12	Turkey	Turkish	Cross-sectional design	120 caregivers of chronic illnesses patients	42.48 ± 12.08	2021
Liu et al. (2023)	AGS-SC	China	Chinese	Cross-sectional design	300 caregivers of stroke patients	45.8 ± 11.1	2022

NR, not reported; PROMs, patient-reported outcome measures; AGS, Anticipatory Grief Scale; MM-CGI, Marwit-Meuser caregiver grief inventory; PGAC, preparatory grief in advanced cancer; MM-CGI-SF, Marwit-Meuser caregiver grief inventory short form; TIGDS, Terminally III Grief or Depression Scale; MM-CGI-CCS, the MM-CGI Childhood Cancer Scale; MM-CGI-CCPS, the MM-CGI Childhood Cerebral Palsy Scale; CGS, Caregiver Grief Scale; C-PGAC, Chinese Version of Preparatory Grief in Advanced Cancer Patients Scale; C-AGS, Chinese Version of Anticipatory Grief Scale; PG-12, prolonged grief disorder questionnaire–predeath; C-MM-CGI-SF, Chinese Version of the Marwit–Meuser caregiver grief inventory–short form; MM-CGI-BF, 6-item Caregiver Grief Scale; C-MM-CGI, Chinese version of the Marwit-Meuser caregiver grief inventory; T-MM-CGI-SF, Turkish version of Marwit-Meuser caregiver grief inventory-short form; AGS-13, Anticipatory Grief Scale-13; CGQ, caregiver grief questionnaire; T-PG-12, Turkish version of prolonged grief disorder-caregiver form; AGS-SC, Anticipatory Grief Scale for stroke caregivers.

### 3.3 Basic characteristics of AG measurement tools

As shown in Table 2, among the 20 included studies, 13 reported the characteristics of AG measurement tools. Two instruments were designed for CIs patients, and 11 for informal caregivers of CIs patients. Of the 13 PROMs, 11 PROMs (Marwit and Meuser, 2002, 2005; Mystakidou et al., 2005; Periyakoil et al., 2005; Al-Gamal et al., 2009; Al-Gamal and Long, 2014; Meichsner et al., 2016; Coelho et al., 2017; Cheng et al., 2019; Holm et al., 2019; Liu et al., 2023) comprised multiple subscales (ranging from 2 to 7 subscales), while one PROM (Liew and Yap, 2018) were unidimensional. In addition, one PROM (Xin, 2017) was originally developed as unidimensional but was validated as multidimensional in its cross-cultural adaptation. The total number of items in the 13 PROMs varied from 6 to 50. The MM-CGI has the largest number of items (50) (Marwit and Meuser, 2002). Four PROMs (Theut et al., 1991; Marwit and Meuser, 2002; Marwit and Meuser, 2005; Coelho et al., 2017) were available in multiple languages. Regarding reliability reporting, 12 PROMs provided Cronbach’s *α* coefficients, and 1 PROM reported a Kappa coefficient. Overall, existing AG PROMs demonstrate substantial heterogeneity in terms of structural dimensions, number of items, available language versions, and reliability metrics.

**Table 2:** Patient-reported outcome measure characteristics.

PROMs	References	Cronbach’s alpha/Factor loadings	Mode of administration	Item number and Subscale	Range of scores	Original language
AGS	Theut et al. (1991)	0.840	Self-report questionnaire	27 items	5-point Likert scale/Total score: 27–135	English
C-AGS	Xin (2017)	0.896/Factor1-7: 0.619 ~ 0.856	Self-report questionnaire	27 items/7 subscales ①	5-point Likert scale/Total score: 27–135	English
PGAC	Mystakidou et al. (2005)	0.838/Factor1-7:0.618; 0.623; 0.714; 0.737;0.727; 0.694; 0.730	Self-report questionnaire	31 items/7 subscales ②	4-point Likert scale/Total score: 0–93	Greek
C-PGAC	Xin (2017)	0.919/Factor1-7: 0.533; 0.740; 0.773; 0.716; 0.753; 0.775; 0.926	Self-report questionnaire	31 items/7 subscales ②	4-point Likert scale/Total score: 0–93	Greek
MM-CGI	Marwit and Meuser (2002)	0.960/Factor 1: 0.93, Factor 2: 0.90, Factor 3: 0.91	Self-report questionnaire	50 items/3 subscales ③	5-point Likert scale/Total score: 50–250	English
MM-CGI	Liew (2016)	0.970/Factor 1: 0.94, Factor 2: 0.92, Factor 3: 0.89	Self-report questionnaire	50 items/3 subscales ③	5-point Likert scale/Total score: 50–250	English
C-MM-CGI	Liew et al. (2018)	0.830	Self-report questionnaire	50 items/3 subscales ③	5-point Likert scale/Total score: 50–250	English
MM-CGI-SF	Marwit and Meuser (2005)	Factor 1: 0.83, Factor 2: 0.80, Factor 3: 0.80	Self-report questionnaire	18 items/3 subscales ③	5-point Likert scale/Total score: 18–90	English
C-MM-CGI-SF	Chan et al. (2017)	0.940/Factor 1: 0.91, Factor 2: 0.88, Factor 3: 0.86	Self-report questionnaire	18 items/3 subscales ③	5-point Likert scale/Total score:18–90	English
T-MM-CGI-SF	Ar-Karci and Karanci (2019)	0.920/Factor 1: 0.88, Factor 2: 0.82, Factor 3: 0.82	Self-report questionnaire	18 items/3 subscales ③	5-point Likert scale/Total score:18–90	English
MM-CGI-SF	Gilsenan et al. (2022)	Factor 1: 0.80, Factor 2: 0.85, Factor 3: 0.85	Self-report questionnaire	18 items/3 subscales ③	5-point Likert scale/Total score:18–90	English
TIGDS	Periyakoil et al. (2005)	Kappa: 0.42	Self-report questionnaire	42 items/2 subscales ④	5-point Likert scale/Total score: 42–210	English
MM-CGI CCS	Al-Gamal et al. (2009)	0.950/Factor 1: 0.91, Factor 2: 0.90, Factor 3: 0.86	Self-report questionnaire	50 items/3 subscales ③	5-point Likert scale/Total score: 50–250	English
MM-CGI CCPS	Al-Gamal and Long (2014)	0.960/Factor 1: 0.93, Factor 2: 0.91, Factor 3: 0.76	Self-report questionnaire	50 items/3 subscales ③	point Likert scale/Total score: 50–250	English
PG-12	Coelho et al. (2017)	0.846/Factor 1: 0.78, Factor 2: 0.97, Factor 3: 0.85	Self-report questionnaire	12 items/3 subscales ⑤	5-point Likert scale/Total score: 12–60	English
T-PG-12	Önal et al. (2023)	0.850/Factor 1: 0.59, Factor 2: 0.42, Factor 3: 0.53	Self-report questionnaire	12 items/3 subscales ⑤	5-point Likert scale/Total score: 12–60	English
CGS	Meichsner et al. (2016)	0.890/Factor 1: 0.810, Factor 2: 0.890, Factor 3: 0.820, Factor 4: 0.670	Self-report questionnaire	11 items/4 subscales ⑥	5-point Likert scale/Total score: 21–105	Greman
MM-CGI-BF	Liew and Yap (2018)	0.840	Self-report questionnaire	6 items	5point Likert scale/Total score: 6–30	English
AGS-13	Holm et al. (2019)	Factor 1: 0.83, Factor 2: 0.840	Self-report questionnaire	13 items/2 subscales ⑦	5-point Likert scale/Total score: 13–65	English
CGQ	Cheng et al. (2019)	0.900/Factor 1: 0.99, Factor 2: 0.84	Self-report questionnaire	11 items/2 subscales ⑧	5-point Likert scale/Total score: 11–55	Chinese
AGS-SC	Liu et al. (2023)	0.842/Factor 1–7: 0.527 ~ 0.747	Self-report questionnaire	24 items/7 subscales ①	5-point Likert scale/Total score: 24–120	English

AD, alzheimer’s disease; ① C-AGS, 7 subscales contains: anger, guilt, anxiety, irritability, sadness, feelings of loss, and decreased ability to function; ② PGAC, 7 subscales contains: self-consciousness, disease adjustment, sadness, anger, religious comfort, somatic symptoms, perceived social support; ③ MM-CGI, MM-CGI-SF, MM-CGI-CCS, MM-CGI-CCPS, 3 subscales contains: personal sacrifice burden, heartfelt sadness and longing, worry and felt isolation; ④ TIGDS, 2 subscales contains: preparatory grief, depression; ⑤ PG-12, 3 subscales contains: separation distress, emotional and cognitive symptoms; ⑥ CGS, 4 subscales contains: emotional pain, relational loss, absolute loss, and acceptance of loss; ⑦ AGS-13, 2 subscales contains: behavioral reactions and motional reactions; ⑧ CGQ, 2 subscales contains: relational deprivation and emotional pain.

### 3.4 Methodological quality and measurement properties

Among the 20 included studies, the number of psychometric properties examined in each study ranged from 3 to 6, with 12 studies investigating five or more properties (Marwit and Meuser, 2002; Mystakidou et al., 2005; Periyakoil et al., 2005; Meichsner et al., 2016; Coelho et al., 2017; Xin, 2017; Liew and Yap, 2018; Ar-Karci and Karanci, 2019; Cheng et al., 2019; Holm et al., 2019; Önal et al., 2023; Liu et al., 2023). All studies consistently reported on content validity, internal consistency, and hypothesis testing analysis results. However, these studies were lacking in information regarding result validity, cross-cultural validity, stability, and criterion validity, and did not provide data on measurement error and responsiveness.

#### 3.4.1 Content validity

As shown in Tables 3, 4, the content validity of 20 studies was evaluated. 9 studies (Marwit and Meuser, 2005; Mystakidou et al., 2005; Periyakoil et al., 2005; Al-Gamal et al., 2009; Chan et al., 2017; Coelho et al., 2017; Xin, 2017; Cheng et al., 2019; Liu et al., 2023) ensured the reliability of content validity by obtaining the opinions of consulting experts or test subjects regarding the constructs of the measurement tools, and selected appropriate analysis methods. Therefore, the methodological quality was rated as “adequate.” 11 studies were rated as “doubtful,” among which three studies (Theut et al., 1991; Al-Gamal and Long, 2014; Meichsner et al., 2016) did not conduct pre-experiments, and eight studies (Marwit and Meuser, 2002; Liew, 2016; Liew and Yap, 2018; Liew et al., 2018; Ar-Karci and Karanci, 2019; Holm et al., 2019; Gilsenan et al., 2022; Önal et al., 2023) had unclear descriptions of concepts or methodologies. Based on the assessment results of each study in terms of comprehensiveness, relevance, and understandability. Two PROMs, including the Marwit-Meuser Caregiver Grief Inventory Short Form (MM-CGI-SF) and Prolonged Grief Disorder Questionnaire–Predeath (PG-12) were rated as “sufficient (+).” eleven PROMs, including the Preparatory Grief in Advanced Cancer (PGAC), Terminally III Grief or Depression Scale (TIGDS), Marwit-Meuser Caregiver Grief Inventory (MM-CGI), MM-CGI Childhood Cancer Scale (MM-CGI-CCS), Anticipatory Grief Scale (AGS), Anticipatory Grief Scale-13 (AGS-13), Anticipatory Grief Scale for Stroke Caregivers (AGS-SC), 6-item Caregiver Grief Scale (MM-CGI-BF), MM-CGI Childhood Cerebral Palsy Scale (MM-CGI-CCPS), Caregiver Grief Scale (CGS), and Caregiver Grief Questionnaire (CGQ) were rated as “indeterminate (?)” due to insufficient evidence.

**Table 3:** Methodological quality assessment.

Target population	Reference	PROMs	Content validity	Structural validity	Internal consistency	Cross-cultural validity	Reliability	Measurement error	Criterion validity	Hypothesis testing	Responsiveness
Patients	Mystakidou et al. (2005)	PGAC	A	A	V	NA	I	NA	NA	D	NA
	Xin (2017)	C-PGAC	A	A	V	I	NA	NA	NA	A	NA
	Periyakoil et al. (2005)	TIGDS	A	A	V	NA	V	NA	V	D	NA
Caregivers	Theut et al. (1991)	AGS	D	NA	V	NA	NA	NA	NA	A	NA
	Xin (2017)	C-AGS	A	A	V	I	NA	NA	NA	A	NA
	Marwit and Meuser (2002)	MM-CGI	D	D	V	NA	NA	NA	NA	A	NA
	Liew (2016)	MM-CGI	D	D	V	I	NA	NA	NA	A	NA
	Liew et al. (2018)	C-MM-CGI	D	NA	V	NA	V	NA	NA	A	NA
	Marwit and Meuser (2005)	MM-CGI-SF	A	A	V	NA	NA	NA	NA	A	NA
	Chan et al. (2017)	C-MM-CGI-SF	A	D	V	NA	NA	NA	NA	A	NA
	Gilsenan et al. (2022)	MM-CGI-SF	D	V	V	NA	NA	NA	NA	A	NA
	Ar-Karci and Karanci (2019)	T-MM-CGI-SF	D	D	V	I	D	NA	NA	A	NA
	Coelho et al. (2017)	PG-12	A	D	V	I	NA	NA	NA	A	NA
	Önal et al. (2023)	T-PG-12	D	D	V	NA	V	NA	NA	A	NA
	Al-Gamal et al. (2009)	MM-CGI-CCS	A	D	V	NA	NA	NA	NA	D	NA
	Al-Gamal and Long (2014)	MM-CGI-CCPS	D	D	V	NA	NA	NA	NA	A	NA
	Meichsner et al. (2016)	CGS	D	V	V	NA	V	NA	NA	A	NA
	Liew and Yap (2018)	MM-CGI-BF	D	NA	V	I	V	NA	A	A	NA
	Holm et al. (2019)	AGS-13	D	A	V	I	NA	NA	NA	A	NA
	Cheng et al. (2019)	CGQ	A	A	V	NA	V	NA	NA	A	NA
	Liu et al. (2023)	AGS-SC	A	V	V	I	I	NA	NA	NA	NA

NA, not applicable; A, adequate; V, very good; D, doubtful; I, inadequate.

**Table 4:** Rating of the measurement properties of the instruments.

Target population	Reference	PROMs	Structural validity	Internal consistency	Cross-cultural validity	Reliability	Measurement error	Criterion validity	Hypothesis testing	Responsiveness
Patients	Mystakidou et al. (2005)	PGAC	+	+	NA	-	NA	NA	-	NA
	Xin (2017)	C-PGAC	+	+	-	NA	NA	NA	+	NA
		Overall rating	+	-	-	-	NA	NA	-	NA
	Periyakoil et al. (2005)	TIGDS	?	+	NA	+	NA	+	-	NA
Caregivers	Theut et al. (1991)	AGS	NA	+	NA	NA	NA	NA	+	NA
	Xin (2017)	C-AGS	+	+	-	NA	NA	NA	?	NA
		Overall rating	+	?	-	NA	NA	NA	-	NA
	Marwit and Meuser (2002)	MM-CGI	?	+	NA	NA	NA	NA	+	NA
	Liew (2016)	MM-CGI	?	+	-	NA	NA	NA	+	NA
	Liew et al. (2018)	C-MM-CGI	NA	+	NA	+	NA	NA	-	NA
		Overall rating	?	-	-	+	NA	NA	+	NA
	Marwit and Meuser (2005)	MM-CGI-SF	+	+	NA	NA	NA	NA	+	NA
	Chan et al. (2017)	C-MM-CGI-SF	?	+	NA	NA	NA	NA	+	NA
	Gilsenan et al. (2022)	MM-CGI-SF	+	+	NA	NA	NA	NA	-	NA
	Ar-Karci and Karanci (2019)	T-MM-CGI-SF	?	+	-	?	NA	NA	+	NA
		Overall rating	+	?	-	?	NA	NA	+	NA
	Coelho et al. (2017)	PG-12	?	+	-	NA	NA	NA	+	NA
	Önal et al. (2023)	T-PG-12	?	+	NA	+	NA	NA	+	NA
		Overall rating	?	+	-	+	NA	NA	+	NA
	Al-Gamal et al. (2009)	MM-CGI-CCS	?	+	NA	NA	NA	NA	-	NA
	Al-Gamal and Long (2014)	MM-CGI-CCPS	?	+	NA	NA	NA	NA	+	NA
	Meichsner et al. (2016)	CGS	+	+	NA	-	NA	NA	+	NA
	Liew and Yap (2018)	MM-CGI-BF	NA	+	-	+	NA	+	+	NA
	Holm et al. (2019)	AGS-13	?	+	-	NA	NA	NA	+	NA
	Cheng et al. (2019)	CGQ	+	+	NA	+	NA	NA	+	NA
	Liu et al. (2023)	AGS-SC	+	+	-	-	NA	NA	NA	NA

NA, not applicable; +, sufficient; −, insufficient;?, indeterminate.

#### 3.4.2 Internal structure

The internal structure (including structural validity, internal consistency, and cross-cultural validity) was presented in Tables 3, 4. Structural validity was assessed in 16 studies. Three studies (Coelho et al., 2017; Cheng et al., 2019; Önal et al., 2023) employed confirmatory factor analysis (CFA), four studies (Marwit and Meuser, 2005; Mystakidou et al., 2005; Xin, 2017; Holm et al., 2019) utilized EFA, and 3 studies (Meichsner et al., 2016; Gilsenan et al., 2022; Liu et al., 2023) applied both CFA and EFA, and two studies (Marwit and Meuser, 2002; Ar-Karci and Karanci, 2019) used principal component analysis (PCA). Four studies did not clearly report their validation methods (Al-Gamal et al., 2009; Al-Gamal and Long, 2014; Liew, 2016; Chan et al., 2017). Methodological quality assessment revealed that three studies (Meichsner et al., 2016; Gilsenan et al., 2022; Liu et al., 2023) were rated as “very good,” five studies (Marwit and Meuser, 2005; Mystakidou et al., 2005; Xin, 2017; Cheng et al., 2019; Holm et al., 2019) as “adequate,” and eight studies (Marwit and Meuser, 2002; Ar-Karci and Karanci, 2019; Coelho et al., 2017; Önal et al., 2023) as “doubtful.” Structural validity Psychological attribute results: The structural validity of six PROMs (AGS, AGS-SC, PGAC, MM-CGI-SF, CGS, and CGQ) was rated as “sufficient (+),” and the structural validity of six PROMs (AGS-13, MM-CGI, MM-CGI-BF, MM-CGI-CCS, MM-CGI-CCPS, and PG-12) was rated as “indeterminate(?)” due to insufficient evidence or unclear description of methods.

Internal consistency was assessed in all studies, with methodological quality rated as “very good” across all evaluations. 19 studies (Theut et al., 1991; Marwit and Meuser, 2002; Marwit and Meuser, 2005; Mystakidou et al., 2005; Al-Gamal et al., 2009; Al-Gamal and Long, 2014; Liew, 2016; Meichsner et al., 2016; Chan et al., 2017; Coelho et al., 2017; Xin, 2017; Liew and Yap, 2018; Liew et al., 2018; Ar-Karci and Karanci, 2019; Cheng et al., 2019; Holm et al., 2019; Gilsenan et al., 2022; Önal et al., 2023; Liu et al., 2023) calculated and reported Cronbach’s *α* coefficients, while one study (Periyakoil et al., 2005) reported Cohen’s kappa coefficient (*κ*) with 95% *CI*. Regarding internal consistency psychometric properties, 20 studies (Theut et al., 1991; Marwit and Meuser, 2002; Marwit and Meuser, 2005; Al-Gamal et al., 2009; Al-Gamal and Long, 2014; Liew, 2016; Meichsner et al., 2016; Chan et al., 2017; Coelho et al., 2017; Xin, 2017; Liew and Yap, 2018; Liew et al., 2018; Ar-Karci and Karanci, 2019; Cheng et al., 2019; Holm et al., 2019; Gilsenan et al., 2022; Önal et al., 2023; Liu et al., 2023; Mystakidou et al., 2005; Periyakoil et al., 2005) demonstrated Cronbach’s *α* coefficients that met the threshold and were therefore rated “sufficient (+).”

Cross-cultural validity was assessed in seven studies that reported on the cross-cultural adaptation and psychometric evaluation of AG PROMs in non-English–speaking populations. One study (Xin, 2017) included both the Chinese Version of Anticipatory Grief Scale (C-AGS) and Chinese Version of Preparatory Grief in Advanced Cancer Patients Scale (C-PGAC), which underwent forward–backward translation, expert review, cognitive interviewing, and comprehensive validation in a Chinese sample. Similarly, five studies (Coelho et al., 2017; Liew and Yap, 2018; Ar-Karci and Karanci, 2019; Holm et al., 2019; Liu et al., 2023) followed standard adaptation procedures and evaluated the psychometric properties of the instruments within their respective cultural contexts. However, all analyses were confined to single-culture samples and did not include comparisons with the original English versions or other language versions using measurement invariance testing. Notably, one study (Liew, 2016) reported a significant difference in MM-CGI scores between Asian and U.S. caregivers (*p* < 0.001); however, without conducting multi-group confirmatory factor analysis or differential item functioning analysis, potential measurement bias could not be ruled out, and thus the observed difference cannot be interpreted as a true cultural difference. In summary, although these studies provide strong support for the local applicability of the instruments, none performed formal cross-cultural validity testing. Therefore, the methodological quality of these studies regarding cross-cultural validity was rated as “inadequate.” The evidence for this measurement property was rated as “insufficient (−).”

#### 3.4.3 Assessment of quality and results for the remaining measurement properties

Tables 3, 4 present the assessment results of reliability, criterion validity, and hypothesis testing. Reliability was assessed in 9 studies. One study (Periyakoil et al., 2005) calculated the kappa statistic. Five studies (Meichsner et al., 2016; Liew and Yap, 2018; Liew et al., 2018; Cheng et al., 2019; Önal et al., 2023) reported an intraclass correlation coefficient (ICC) ≥ 0.8, indicating good test–retest reliability, and were rated as “very good” in methodological quality, one study (Ar-Karci and Karanci, 2019) that reported ICC without specifying the retest interval was rated as “doubtful,” one study (Liu et al., 2023) with an ICC below the threshold was rated as “inadequate.” Nine studies assessed test–retest reliability intervals. Five studies (Periyakoil et al., 2005; Liew and Yap, 2018; Liew et al., 2018; Cheng et al., 2019; Önal et al., 2023) that met the 14-day retest criterion were rated as “sufficient (+)”; three studies (Mystakidou et al., 2005; Meichsner et al., 2016; Liu et al., 2023) that failed to meet this standard were rated as “insufficient (−);” and one study (Ar-Karci and Karanci, 2019) with an unspecified interval duration was rated as “indeterminate (?).” The synthesized results of reliability measurement properties indicated that five PROMs (TIGDS, MM-CGI, PG-12, MM-CGI-BF, and CGQ) were rated as “sufficient (+).” Three PROMs (CGS, PGAC, and AGS-SC) were classified as “insufficient (−),” and one instrument (MM-CGI-SF) was rated as “indeterminate (?).”

20 studies reported hypothesis testing results. 19 studies (Theut et al., 1991; Marwit and Meuser, 2002, 2005; Mystakidou et al., 2005; Periyakoil et al., 2005; Al-Gamal et al., 2009; Al-Gamal and Long, 2014; Liew, 2016; Meichsner et al., 2016; Chan et al., 2017; Coelho et al., 2017; Xin, 2017; Liew and Yap, 2018; Liew et al., 2018; Ar-Karci and Karanci, 2019; Cheng et al., 2019; Holm et al., 2019; Gilsenan et al., 2022; Önal et al., 2023) employed convergent or discriminant validity analyses to examine the target instruments against generic comparator scales, though the applicability of these scales to the specific population remains unclear. 16 studies (Theut et al., 1991; Marwit and Meuser, 2002, 2005; Al-Gamal and Long, 2014; Liew, 2016; Meichsner et al., 2016; Chan et al., 2017; Coelho et al., 2017; Xin, 2017; Liew and Yap, 2018; Liew et al., 2018; Ar-Karci and Karanci, 2019; Cheng et al., 2019; Holm et al., 2019; Gilsenan et al., 2022; Önal et al., 2023) were therefore rated as “adequate.” Three studies (Mystakidou et al., 2005; Periyakoil et al., 2005; Al-Gamal et al., 2009) did not adequately address the measurement attributes of the comparison tools and were rated as “doubtful.” The overall result of the measurement attributes of the hypothesis test: eight PROMs (AGS-13, MM-CGI, MM-CGI-SF, PG-12, MM-CGI-CCPS, CGS, MM-CGI-BF, and CGQ) have a consistent correlation with the hypothesis, and are rated as “sufficient (+)”, four PROMs (MM-CGI-CCS, PGAC, TIGDS, and AGS) have unclarified hypothesis test information and are rated as “insufficient (−).”

Only two studies (Periyakoil et al., 2005; Liew and Yap, 2018) reported on criterion validity. Both studies employed receiver operating characteristic (ROC) curve analysis to assess the scale’s criterion validity, which is its ability to discriminate between groups in relation to a gold-standard reference, and reported the corresponding sensitivity and specificity. One study (Periyakoil et al., 2005) (TIGDS), which used clinical consensus as the gold standard, was rated as “very good” and the measurement attribute was “sufficient (+).” The other study (Liew and Yap, 2018) (MM-CGI-BF) was rated as having “adequate” methodological quality, and the measurement attribute was also “sufficient (+).”

#### 3.4.4 Meta-analysis results

**Figure 2:**
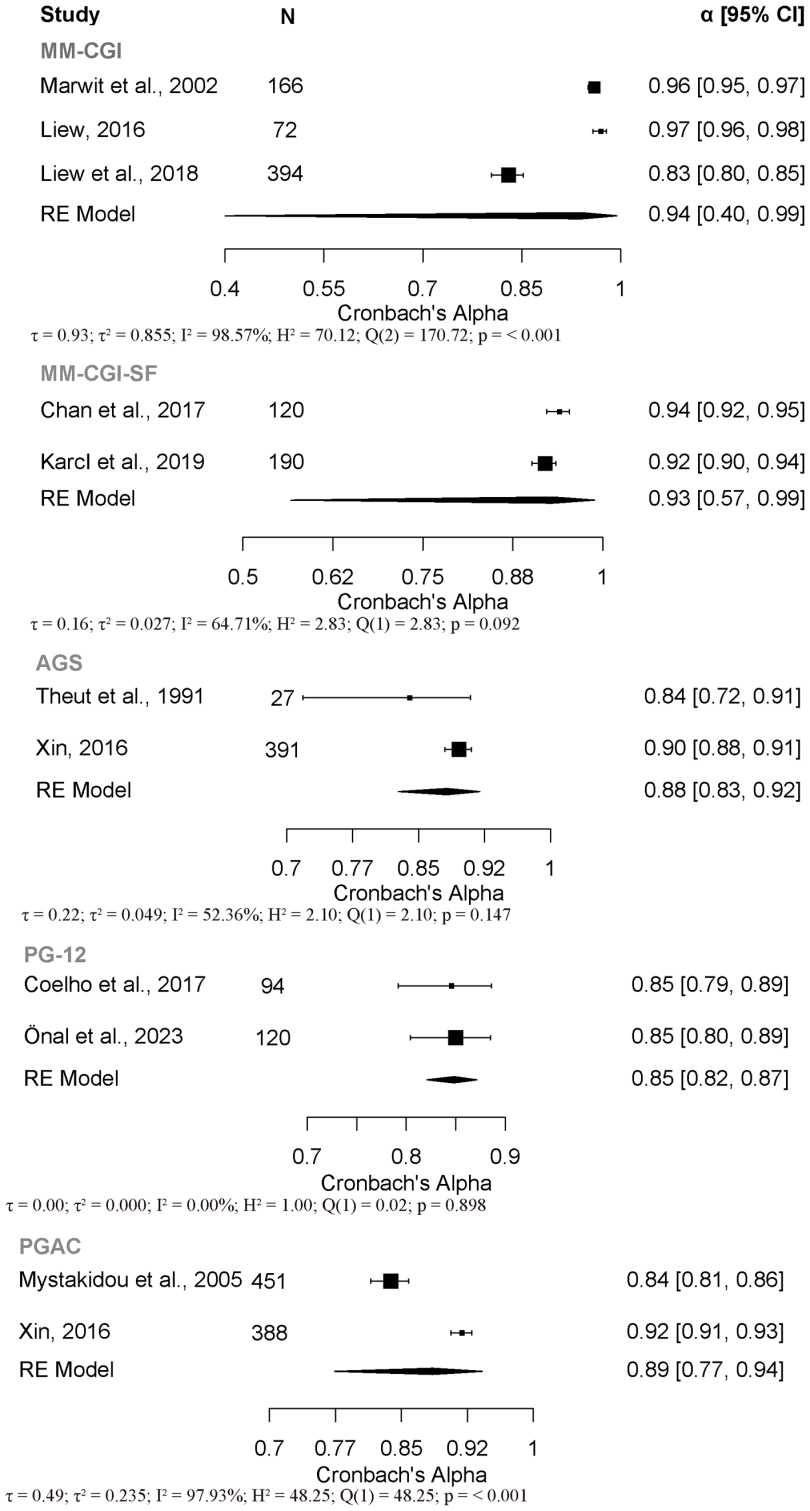
Forest plot of internal consistency coefficients for the AG PROMs total score. MM-CGI, Marwit-Meuser caregiver grief inventory; MM-CGI-SF, Marwit-Meuser caregiver grief inventory short form; AGS, Anticipatory Grief Scale; PG-12, Prolonged Grief Scale; PGAC, preparatory grief in advanced cancer; *α*, Cronbach’s alpha; 95% CI, 95% confidence interval.

This study employed a RG meta-analysis to synthesize the Cronbach’s *α* coefficients of five AG PROMs to evaluate their internal consistency (Figure 2). For PG-12 (*k* = 2, *N* = 214), the pooled *α* coefficient was 0.85 (95% *CI*, 0.82, 0.87), with no observed heterogeneity [*I^2^* = 0%, *H^2^* = 1.00, *Q*(1) = 0.02, *p* = 0.898]. This indicates stable internal consistency across studies, and it was therefore rated as “sufficient (+).” AGS (*k* = 2, *N* = 418), the pooled alpha coefficient was 0.88 (95% *CI*, 0.83, 0.92), but moderate heterogeneity was present [*I^2^* = 52.36%, *H^2^* = 2.10, *Q*(1) = 2.10, *p* = 0.147]. Due to the limited number of studies, the stability of its internal consistency remains questionable, leading to a rating of “indeterminate (?).” For MM-CGI-SF (*k* = 2, *N* = 310), the pooled *α* coefficient was 0.93, but the confidence interval was very wide (95% *CI*, 0.57, 0.99), and moderate heterogeneity was observed [*I^2^* = 64.71%, *H^2^* = 2.83, *Q*(1) = 2.83, *p* = 0.092]. The limited evidence and high uncertainty make it difficult to draw a firm conclusion about its internal consistency, so it was rated as “indeterminate (?).” The analyses for MM-CGI [*k* = 3, *N* = 632, *I^2^* = 98.57%, *H^2^* = 70.12, *Q*(2) = 170.72, *p* < 0.001] and PGAC [*k* = 2, *N* = 839, *I^2^* = 97.93%, *H^2^* = 48.25, *Q*(1) = 48.25, *p* < 0.001] both showed very high heterogeneity, indicating that the internal consistency of MM-CGI and PGAC lacks robustness across studies. Therefore, both were rated as “insufficient (−).”

### 3.5 Measurement attributes of the scale synthesize results and recommendations

Table 5 summarizes the evidence and recommendation grades for 13 PROMs. The evidence quality was downgraded based on four key factors of the modified GRADE approach (risk of bias, inconsistency, indirectness, and imprecision), with the overall certainty of evidence ranging from “high” to “very low.” The evidence grading results showed that two PROMs (PG-12 and MM-CGI-SF) were rated “high” for content validity, while eight PROMs (PG-12, CGS, CGQ, MM-CGI-CCS, MM-CGI-BF, MM-CGI-CCPS, AGS-SC, and AGS-13) were rated “high” for internal consistency. Regarding recommendation grades, the PG-12 was classified as grade A, eleven PROMs (MM-CGI-SF, TIGDS, PGAC, CGS, CGQ, MM-CGI, MM-CGI-BF, MM-CGI-CCS, AGS-SC, AGS and AGS-13) as grade B, and MM-CGI-CCPS as grade C.

**Table 5:** Quality of evidence for each measurement property.

Target population	Recommand	PROMs	Content validity	Structural validity	Internal consistency	Cross-cultural validity	Reliability	Measurement error	Criterion validity	Hypothesis testing	Responsiveness
Patients	B	TIGDS	M	M	M	NA	H	NA	H	M	NA
	B	PGAC	M	H	L	L	L	NA	NA	M	NA
Caregivers	A	PG-12	H	M	H	L	M	NA	NA	H	NA
	B	MM-CGI-SF	H	M	M	L	M	NA	NA	M	NA
	B	CGS	M	H	H	NA	H	NA	NA	H	NA
	B	CGQ	M	M	H	NA	H	NA	NA	H	NA
	B	MM-CGI-CCS	M	M	H	NA	NA	NA	NA	H	NA
	B	MM-CGI	M	M	L	L	M	NA	NA	H	NA
	B	MM-CGI-BF	L	NA	H	L	M	NA	H	H	NA
	B	AGS-SC	M	H	H	L	M	NA	NA	NA	NA
	B	AGS-13	M	M	H	L	NA	NA	NA	H	NA
	B	AGS	L	M	M	L	NA	NA	NA	M	NA
	C	MM-CGI-CCPS	L	M	H	NA	NA	NA	NA	H	NA

NA, not applicable; H, high; M, moderate; L, low; VL, very low.

## 4 Discussion

This review aims to critically examine the measurement tools for AG in patients with CIs and their informal caregivers according to the COSMIN guidelines and to recommend the most effective AG PROMs. The focus is on the reliability, validity, responsiveness, and interpretability of AG measurement tools.

Evaluation of 13 AG PROMs revealed distinct strengths and limitations in their psychometric properties. While content validity was robust for two PROMs, significant gaps were observed in other critical domains. Cross-cultural validity emerged as a particular weakness, with none of the PROMs meeting adequacy standards. Furthermore, criterion validity was substantially understudied, with evidence available for only two PROMs, and test–retest reliability was adequately established for just five PROMs. Notably, none of the studies reported measurement error or responsiveness data.

Cross-cultural validity was not established for any of the eight PROMs evaluated, as none employed recommended methods, such as multi-group confirmatory factor analysis or cross-cultural differential item functioning analysis, to test measurement invariance. These methodological shortcomings suggest that when these instruments are applied across different countries or cultural contexts, measurement bias may arise, limiting the comparability and generalizability of findings. These methodological shortcomings highlight the impact of cultural factors on measurement accuracy. One study (Liew, 2016) reported higher levels of AG among Asian populations compared to American groups, a difference that may be attributed to the stronger emphasis on familial obligations and collectivist values in many Asian societies (Fan et al., 2022; Liang et al., 2024). Within these cultural contexts, expressing emotions openly is often discouraged to maintain family and social harmony, potentially leading to emotional suppression. Such suppression may not only exacerbate psychological distress but also intensify the experience of AG, thereby increasing susceptibility to depression and anxiety (Wang and Shi, 2013; Yu et al., 2021). Further supporting the role of cultural background, Dehpour and Koffman (2023) observed that individuals from certain ethnic groups, such as Malay populations, also exhibited elevated levels of AG. This suggests that the experience of AG may be more influenced by the individual psychological state and cultural background.

In terms of criterion validity, only two PROMs (TIGDS and MM-CGI-BF) focused on the agreement between the assessment tools and the “gold standard.” This may be due to the absence of a recognized gold standard for assessing AG in patients with CIs and their informal caregivers. Notably, without evidence of criterion validity, it is not possible to determine whether changes in instrument scores truly reflect clinically meaningful changes, leading to unquantifiable systematic bias. Lee et al. (2017) pointed out that in the absence of a gold standard, tools with similar measurement dimensions, multi-dimensional structure, and comprehensive content coverage can be selected as the reference basis for criterion validity evaluation. The TIGDS used the clinical consensus of an interdisciplinary hospice and palliative care team as a reference standard to distinguish AG from depressive symptoms (Periyakoil et al., 2005). Although the standard is a non-traditional quantitative tool, it provides a professional basis for discrimination based on the clinical experience of multidisciplinary experts, and its effect size is high. On the other hand, the COSMIN guidelines clearly suggest that only when the simplified version of the scale is compared with the full version of the scale, the full version of the scale can be considered as the “gold standard” for criterion validity assessment (Shi et al., 2021). In the study, the MM-CGI-BF was used to assess AG of informal caregivers of patients with CIs (Liew and Yap, 2018), and the original MM-CGI scale was used as the reference standard. The results showed that there was a strong correlation between the two, thus providing preliminary support for criterion-related validity.

Content validity is an important indicator for evaluating the consistency between the content of AG measurement tool and the target concept, and it is also one of the most crucial measurement attributes in the process of scale development (Peng et al., 2020). For the seven PROMs (AGS, MM-CGI, MM-CGI-SF, PG-12, MM-CGI-CCPS, MM-CGI-BF, CGS, and AGS-13), the cognitive interviews relied mainly on expert input and paid little attention to feedback from the target population regarding their understanding of, feelings about, or suggestions for the items. Although item authority and scientific grounding are important, failing to systematically assess how well the target population understands and accepts the items may widen the cognitive gap between researchers and respondents, thereby increasing measurement error during data collection (Mokkink et al., 2024). Moreover, methodological shortcomings commonly occur during cognitive interviews or pilot testing. Common problems include: lack of cognitive interviews during the development process, conducting only quantitative surveys without combining cognitive interviews, lack of pre-experimental studies, and unclear description of the analysis methods. These issues reduce transparency in content validity development and limit study reproducibility (Huang et al., 2025). More importantly, they weaken the instrument’s reliability across three key dimensions: comprehensiveness, relevance, and comprehensibility, thereby reducing its clinical usefulness and measurement accuracy.

Stability reflects the degree of consistency in the results obtained from multiple tests on the same group of subjects (Peng et al., 2020). The retest interval, as a key evaluation indicator, is related to the measured construct, the target population, and the environment (Han et al., 2023). Short intervals should be avoided (due to the memory effect) or long intervals (due to changes in clinical conditions), to ensure a relatively stable clinical state (Peng et al., 2020). However, the retest intervals in this study ranged from 3 days to 6 months, which may result in insufficient consistency when the same instrument is applied at different time points, thereby reducing the reliability of longitudinal follow-up and repeated measurements. Although the COSMIN guidelines provide reporting norms for retest intervals, AG is a dynamic process. Therefore, when conducting the assessment, it is necessary to clearly define the conditions for retesting and the basis for time selection, control the measurement environment and consistency, reduce the influence of memory effects and structural changes, and improve the applicability and reliability of the scales (Peng et al., 2020; Zhao et al., 2025).

Implication and future research recommendation AG constitutes a significant psychological burden for patients with CIs and their informal caregivers, yet its clinical importance remains underrecognized. Although the Diagnostic and Statistical Manual of Mental Disorders (DSM-5) has incorporated related concepts such as persistent complex bereavement disorder (PCBD), AG itself has not been formally included in major diagnostic systems or clinical guidelines. This omission has contributed to a widespread lack of standardized assessment in routine practice. For researchers and clinicians seeking to evaluate AG using brief self-report instruments, we recommend the PGAC as a preferred tool for assessing AG in patients with CIs, and the PG-12 and MM-CGI-SF for evaluating AG in informal caregivers of CIs patients. Structured assessment tailored to specific populations shows promise for early identification of unmet psychosocial needs and timely intervention, thereby supporting more comprehensive and person-centered CI management.

We propose the following structured approach to advance the development of AG PROMs: For currently low-evidence, B-grade AG PROMs, systematic efforts are needed to upgrade them to COSMIN A-grade standards. First, instruments with insufficient or unclear structural validity evidence should undergo CFA in target populations to provide statistical support for their unidimensional or multidimensional structure. Second, missing content validity evidence should be addressed by revisiting the instrument development process through cognitive interviews or Delphi expert consensus methods. Third, criterion validity studies should replace vague or unvalidated reference standards with well-established, psychometrically sound clinical assessments as anchors. Building on these improvements, future development and validation of AG PROMs should follow a higher-quality methodological framework. Researchers should conduct rigorous cross-cultural validation across diverse ethnic and cultural groups to ensure applicability and generalizability in different populations and clinical settings. Reliable “gold standard” criteria selected based on validity, reliability, and expert consensus should be prioritized in criterion-related validity testing. Finally, strict adherence to COSMIN guidelines is essential, integrating qualitative and quantitative approaches and ensuring clear, transparent, and comprehensive reporting of content validity and other measurement properties.

### 4.1 Limitations

Although this study systematically evaluated the psychometric properties of existing AG PROMs, it also has limitations. First, all included studies were cross-sectional, which precluded the assessment of instrument responsiveness, thereby limiting the applicability of these measures in dynamically tracking the evolution of AG. Future studies should use longitudinal designs with repeated assessments to validate the sensitivity of AG instruments to change and to inform the identification of key intervention windows. Second, the evidence base is restricted to CIs patients and their informal caregivers; thus, generalizability to critically ill populations and their informal caregivers remains uncertain. Furthermore, in the RG meta-analysis, the number of studies included for each PROM was limited (*k ≤* 3), which may compromise the stability of the pooled estimates and the statistical power of the heterogeneity tests. Additionally, the search was limited to English and Chinese language publications, which may introduce language bias. Strengths include strict adherence to the COSMIN methodology and inclusion of at least one psychometric property measurement tool to ensure the accuracy of the results.

## 5 Conclusion

This review of 20 studies identified 13 PROMs and their psychometric properties. The PG-12 demonstrated the best psychometric properties for assessing AG of informal caregivers and was highly recommended for relevant research and clinical practice. However, there is currently no instrument that meets the recommended criteria for grade A to assess patient AG. PGAC has moderate-quality evidence in terms of content validity, structural validity, internal consistency, and cross-cultural validity, and is therefore provisionally recommended. However, it exhibits substantial heterogeneity in internal consistency, and the sample sizes used for its development and validation were relatively small, which may introduce potential bias in the results. Therefore, it is recommended that future studies conduct further validation with larger, multicenter samples to comprehensively evaluate its methodological quality and measurement properties. Future research should focus on validating assessment tools for AG in patients with CIs. It also emphasizes validating the applicability of AG measurement tools for patients with CIs and their informal caregivers across diverse cultural contexts and settings, to advance AG research and optimize clinical practice.

## Data Availability

The datasets used or analysed during the current study are available from the corresponding author on reasonable request.
