# Supplementary material for: Patient-reported outcome measures for anticipatory grief: a systematic review
Source: Front Psychol. 2026 Feb 11;17:1709822. doi: 10.3389/fpsyg.2026.1709822 (PMC12932416; doi:10.3389/fpsyg.2026.1709822)
Supplement: Supplementary file 1 [file Supplementary_file_1.docx]

Supplementary Material

# Supplementary file 1: search strategies

**PubMed/Medline**

Search time: 2024-12-10

| **Search** | **Query** | **Items found** |
| --- | --- | --- |
| #1 | preparatory[Title/Abstract] OR preparedness[Title/Abstract] OR pre-loss[Title/Abstract] OR pre-death[Title/Abstract] OR anticipatory[Title/Abstract] | 30,851 |
| #2 | "Grief"[MeSH] OR "griev*"[Title/Abstract] OR "mourn*"[Title/Abstract] OR "bereave*"[Title/Abstract] | 14,918 |
| #3 | "Surveys and Questionnaires"[MeSH] OR "assess*"[Title/Abstract] OR "instrument"[Title/Abstract] OR "measure*"[Title/Abstract] OR "questionnaire"[Title/Abstract] OR "inventory"[Title/Abstract] OR "scale"[Title/Abstract] OR "interview"[Title/Abstract] OR "criteria"[Title/Abstract] | 5,278,961 |
| #4 | "instrumentation"[MeSH Subheading] OR "reproducibility of results"[MeSH Terms] OR "reproducib*"[Title/Abstract] OR "psychometrics"[MeSH Terms] OR "psychometr*"[Title/Abstract] OR "clinimetr*"[Title/Abstract] OR "clinometr*"[Title/Abstract] OR "observer variation"[MeSH Terms] OR "observer variation"[Title/Abstract] OR "reliab*"[Title/Abstract] OR "valid*"[Title/Abstract] OR "coefficient"[Title/Abstract] OR "internal consistency"[Title/Abstract] OR ("cronbach*"[Title/Abstract] OR ("alpha"[Title/Abstract] OR "alphas"[Title/Abstract]))OR "item correlation"[Title/Abstract]) AND ("measurement"[Title/Abstract] OR "measuring"[Title/Abstract] OR "standard error of measurement"[Title/Abstract]) OR ("sensitiv*"[Title/Abstract] OR "responsive*"[Title/Abstract] OR "limit"[Title/Abstract]) AND ("detection"[Title/Abstract]) OR "minimal detectable concentration"[Title/Abstract] OR "interpretab*"[Title/Abstract] | 874,551 |
| #5 | (#1 AND #2 AND #3 AND #4 )  Filters applied: Full text, English, Humans | 293 |
| #6 | ("biography"[Publication Type] OR "case reports"[Publication Type] OR "comment"[Publication Type] OR "directory"[Publication Type] OR "editorial"[Publication Type] OR "festschrift"[Publication Type] OR "interview"[Publication Type] OR "legislation"[Publication Type] OR "letter"[Publication Type] OR "news"[Publication Type] OR "newspaper article"[Publication Type] OR "patient education handout"[Publication Type] OR "consensus development conference"[Publication Type] OR "consensus development conference, nih"[Publication Type] OR "practice guideline"[Publication Type]) NOT ("animals"[MeSH Terms] NOT "humans"[MeSH Terms]) | 1,340,459 |
| #7 | (#5 NOT #6)  Filters applied: Full text, English | 286 |

**EMBASE**

Search time: 2024-12-10

| **Search** | **Query** | **Items found** |
| --- | --- | --- |
| #1 | ('preparatory' OR 'preparedness'/exp OR 'pre-loss' OR 'pre-death' OR 'anticipatory').ab,ti. | 63,294 |
| #2 | ('Grief'/exp OR 'griev*' OR 'mourn*' OR 'bereave*').ab,ti. | 39,003 |
| #3 | ('Surveys and Questionnaires' OR 'assess*' OR 'instrument' OR 'measure*' OR 'questionnaire' OR 'inventory' OR 'scale' OR 'interview' OR 'criteria').ab,ti. | 13,384,356 |
| #4 | ('instrumentation'/exp OR 'reproducibility'/exp OR 'reproducib*':ti,ab OR 'psychometrics'/exp OR 'psychometr*':ti,ab OR 'clinimetr*':ti,ab OR 'clinometr*':ti,ab OR 'observer variation'/exp OR 'observer variation':ti,ab OR 'reliab*':ti,ab OR 'valid*':ti,ab OR 'coefficient':ti,ab OR 'internal consistency':ti,ab OR ('cronbach*':ti,ab OR ('alpha':ti,ab OR 'alphas':ti,ab)) OR 'item correlation':ti,ab) AND ('measurement':ti,ab OR 'measuring':ti,ab OR 'standard error of measurement':ti,ab) OR ('sensitiv*':ti,ab OR 'responsive*':ti,ab OR 'limit':ti,ab) AND (detection:ti,ab) OR 'minimal detectable concentration':ti,ab OR 'interpretab*':ti,ab | 6.726,684 |
| #5 | #1 AND #2 AND #3 AND #4  Limit to ((full text and english language and embase)) | 597 |

**Web of Science Core Collection**

Search time: 2024-12-10

| **Search** | **Query** | **Items found** |
| --- | --- | --- |
| #1 | (TS=((preparatory OR preparedness OR pre-loss OR pre-death OR anticipatory)) | 102,126 |
| #2 | (TS=(griev* OR mourn* OR bereave* OR Grief)) | 51,542 |
| #3 | (TS=assess* OR instrument OR measure* OR questionnaire OR inventory OR scale OR interview OR criteria OR Surveys and Questionnaires)) | 13,927,816 |
| #4 | TS=(instrumentation) OR AB=(instrumentation) OR TI=(instrumentation) OR TS=(reproducib*) OR AB=(reproducib*) OR TI=(reproducib*) OR TS=(psychometr*) OR AB=(psychometr*) OR TI=(psychometr*) OR TS=(clinimetr*) OR AB=(clinimetr*") OR TI=(clinimetr*) OR TS=(clinometr*) OR AB=(clinometr*) OR TI=(clinometr*) OR TS=(observer variation) OR AB=(observer variation) OR TI=(observer variation) OR TS=(reliab*) OR AB=("reliab*") OR TI=(reliab*) OR TS=(valid*) OR AB=(valid*) OR TI=(valid*) OR TS=(coefficient) OR AB=(coefficient") OR TI=(coefficient) OR TS=(internal consistency) OR AB=(internal consistency) OR TI=(internal consistency) OR TS=(cronbach*) OR AB=(cronbach*) OR TI=(cronbach*) OR TS=(alpha) OR AB=(alpha) OR TI=(alpha) OR TS=(alphas) OR AB=(alphas) OR TI=(alphas))) OR TS=(item correlation) OR AB=(item correlation) OR TI=(item correlation)) AND TS=(measurement) OR AB=(measurement) OR TI=(measurement) OR TS=(measuring) OR AB=(measuring) OR TI=(measuring) OR TS=(standard error of measurement) OR AB=(standard error of measurement) OR TI=(standard error of measurement)) OR TS=(sensitiv*) OR AB=(sensitiv*) OR TI=(sensitiv*) OR TS=(responsive*) OR AB=(responsive*) OR TI=(responsive*) OR TS=(limit) OR AB=(limit) OR TI=(limit)) AND TS=(detection) OR AB=(detection) OR TI=(detection)) OR TS=(minimal detectable concentration) OR AB=(minimal detectable concentration) OR TI=(minimal detectable concentration) OR TS=(interpretab*) OR AB=(interpretab*) OR TI=(interpretab*) | 5.613,524 |
| #5 | (#1 AND #2 AND #3 AND #4)  Refined by: Languages: English; Document Types: Articles. | 686 |

**CINAHL (EBSCO)**

Search time: 2024-12-10

| **Search** | **Query** | **Items found** |
| --- | --- | --- |
| #1 | SU (preparatory OR preparedness OR pre-loss OR pre-death OR anticipatory ) | 50,544 |
| #2 | (MH "Grief") OR SU (griev* OR mourn* OR bereave*) | 1,221,512 |
| #3 | (MH "Surveys and Questionnaires) OR SU(assess* OR instrument OR measure* OR questionnaire OR inventory OR scale OR interview OR criteria) | 9,489,941 |
| #4 | (MH "Instrumentation" OR MH "Reproducibility of Results" OR MH "Psychometrics" OR MH "Observer Variation" OR TI instrumentation OR AB instrumentation OR  TI reproducib* OR AB reproducib* OR TI psychometr* OR AB psychometr* OR TI clinimetr* OR AB clinimetr* OR TI clinometr* OR AB clinometr* OR TI observer variation OR AB observer variation OR TI reliab* OR AB reliab* OR TI valid* OR AB valid* OR TI coefficient OR AB coefficient OR TI internal consistency OR AB internal consistency OR TI cronbach* OR AB cronbach* OR TI alpha OR AB alpha OR TI alphas OR AB alphas OR TI item correlation OR AB item correlation) AND (MH "Measurement" OR MH "Sensitivity and Specificity" OR TI measurement OR AB measurement OR TI measuring OR AB measuring OR TI standard error of measurement OR AB standard error of measurement OR TI sensitiv* OR AB sensitiv* OR TI responsive* OR AB responsive* OR TI limit OR AB limit) AND (MH "Detection" OR MH "Interpretation" OR TI detection OR AB detection OR TI minimal detectable concentration OR AB minimal detectable concentration OR TI interpretab* OR AB interpretab*) | 633,701 |
| #5 | #1 AND #2 AND #3 AND #4 | 335 |

**PsycINFO (EBSCO)**

Search time: 2024-12-10

| **Search** | **Query** | **Items found** |
| --- | --- | --- |
| #1 | ab(preparatory OR preparedness OR pre loss OR pre death OR anticipatory) | 23,852 |
| #2 | mainsubject (Grief) OR ab (griev* OR mourn* OR bereave*) | 29,353 |
| #3 | ab(Surveys and Questionnaires OR assess* OR instrument OR measure* OR questionnaire OR inventory OR scale OR interview OR criteria) | 2,648,589 |
| #4 | cl(“Psychometrics & Statistics & ‎Methodology” OR “Research ‎Methods & Experimental Design”) OR instrumentation OR reproducibility of results OR reproducib* OR psychometrics OR psychometr* OR clinimetr* OR clinometr* OR observer variation OR observer variation OR reliab* OR valid* OR coefficient OR internal consistency OR cronbach* OR alpha OR alphas OR item correlation OR measurement OR measuring OR standard error of measurement OR sensitiv* OR responsive* OR limit OR detection OR minimal detectable concentration OR interpretab*  Refined by: Languages: English; Full text  Publication Types: Academic journal, Dissertation; Document Types: Book, Book chapter, article, These. | 562,593 |
| #5 | (#1 AND #2 AND #3 AND #4 ) | 396 |

**Cochrane Library**

Search time: 2024-12-10

| **Search** | **Query** | **Items found** |
| --- | --- | --- |
| #1 | (preparatory OR preparedness OR pre loss OR pre death OR anticipatory):ti,ab,kw | 3,338 |
| #2 | (grief OR grief* OR mourn* OR bereave):ti,ab,kw | 1,308 |
| #3 | (Surveys and Questionnaires OR assess* OR instrument OR measure* OR questionnaire OR inventory OR scale OR interview OR criteria ):ti,ab,kw | 641,065 |
| #4 | (instrumentation OR reproducibility OR reproducib* OR psychometrics OR psychometr* OR clinimetr* OR clinometr* OR observer variation OR observer variation OR reliab* OR valid* OR coefficient OR internal consistency OR cronbach* OR alpha OR alphas OR item correlation OR measurement OR measuring OR standard error of measurement) OR sensitiv* OR responsive* OR limit OR detection OR minimal detectable concentration OR interpretab*):ti,ab,kw | 376,247 |
| #5 | #1 AND #2 AND #3 AND #4 | 54 |

**CNKI**

Search time: 2024-12-10

| **Search** | **Query** | **Items found** |
| --- | --- | --- |
| #1 | SU=预期性悲伤 + 预感性悲伤 + 预期性哀伤 + 预感性哀伤 | 117 |
| #2 | SU=工具 + 问卷 + 量表 | 489,353 |
| #3 | SU = 效度 + 信度 + 克朗巴赫系数 + 组内相关系数 + 结构效度 + 内容效度 + 内部一致性 + 测量属性 | 43,905 |
| #4 | #1 AND #2 AND #3 | 128 |

**WanFang**

Search time: 2024-12-10

| **Search** | **Query** | **Items found** |
| --- | --- | --- |
| #1 | 主题:(预期性悲伤 OR 预感性悲伤 OR 预期性哀伤 OR 预感性哀伤) | 264 |
| #2 | 主题:(工具 OR 问卷 OR 量表) | 2,794,562 |
| #3 | 主题:(信度 or 效度 or 克朗巴赫系数 or 组内相关系数 or 结构效度 or 内容效度 or 内部一致性 or 测量属性) | 3,992,135 |
| #4 | #1 AND #2 AND #3 | 175 |

**China Biology Medicine Database**

Search time: 2024-12-10

| **Search** | **Query** | **Items found** |
| --- | --- | --- |
| #1 | 常用字段:(预期性悲伤OR 预感性悲伤OR 预期性哀伤OR预期性哀伤) | 142 |
| #2 | 常用字段:(量表 or 问卷 or 测量工具) | 2,167,518 |
| #3 | 常用字段:(信度 or 效度 or 克朗巴赫系数 or 组内相关系数 or 结构效度 or 内容效度 or 内部一致性 or 测量属性) | 766,110 |
| #4 | #1 AND #2 AND #3 | 114 |
