# Supplementary material for: Patient-reported outcome measures for anticipatory grief: a systematic review
Source: Front Psychol. 2026 Feb 11;17:1709822. doi: 10.3389/fpsyg.2026.1709822 (PMC12932416; doi:10.3389/fpsyg.2026.1709822)
Supplement: Supplementary file 2 [file Supplementary_file_2.docx]

Supplementary Material

# Supplementary file 2: References of the 20 RG meta-analyses used in the reliability study of the REGEMA checklist

Al-Gamal, E., and Long, T. (2014). The MM-CGI Cerebral Palsy: modification and pretesting of an instrument to measure anticipatory grief in parents whose child has cerebral palsy. J Clin Nurs 23, 1810–1819. doi: 10.1111/jocn.12218

Al-Gamal, E., Long, T., and Livesley, J. (2009). Development of a Modified Instrument to Measure Anticipatory Grieving in Jordanian Parents of Children Diagnosed With Cancer: The Marwit and Meuser Caregiver Inventory Childhood Cancer. Cancer Nursing 32, 211–219. doi: 10.1097/NCC.0b013e31819a2ae4

Ar-Karci, Y., and Karanci, A. N. (2019). Examination of the Psychometric Properties of Marwit-Meuser Caregiver Grief Inventory-Short Form. Turkish Journal of Psychiatry. doi: 10.5080/u23501

Chan, W. C. H., Wong, B., Kwok, T., and Ho, F. (2017). Assessing Grief of Family Caregivers of People with Dementia: Validation of the Chinese Version of the Marwit–Meuser Caregiver Grief Inventory. Health & Social Work 42, 151–158. doi: 10.1093/hsw/hlx022

Cheng, S. T., Ma, D. Y., and Lam, L. C. W. (2019). A brief measure of predeath grief in dementia caregivers: the Caregiver Grief Questionnaire. International Psychogeriatrics 31, 1099–1107. doi: 10.1017/S1041610219000309

Coelho, A., Silva, C., and Barbosa, A. (2017). Portuguese validation of the Prolonged Grief Disorder Questionnaire–Predeath (PG–12): Psychometric properties and correlates. Pall Supp Care 15, 544–553. doi: 10.1017/S1478951516001000

Gilsenan, J., Gorman, C., and Shevlin, M. (2022). Exploratory factor analysis of the caregiver grief inventory in a large UK sample of dementia carers. Aging & Mental Health 26, 320–327. doi: 10.1080/13607863.2020.1839856

Holm, M., Alvariza, A., Fürst, C.-J., Öhlen, J., and Årestedt, K. (2019). Psychometric evaluation of the anticipatory grief scale in a sample of family caregivers in the context of palliative care. Health Qual Life Outcomes 17, 42. doi: 10.1186/s12955-019-1110-4

Liew, T. M. (2016). Applicability of the pre‐death grief concept to dementia family caregivers in Asia. Int J Geriat Psychiatry 31, 749–754. doi: 10.1002/gps.4387

Liew, T. M., and Yap, P. (2018). A Brief, 6-Item Scale for Caregiver Grief in Dementia Caregiving. The Gerontologist. doi: 10.1093/geront/gny161

Liew, T. M., Yap, P., Luo, N., Hia, S. B., Koh, G. C.-H., and Tai, B. C. (2018). Detecting pre-death grief in family caregivers of persons with dementia: measurement equivalence of the Mandarin-Chinese version of Marwit-Meuser caregiver grief inventory. BMC Geriatr 18, 114. doi: 10.1186/s12877-018-0804-5

Liu, X., Zhou, Y. M., Xu, H. L., Peng, J. Y., Xie, Z. S., and Xing, L. M. (2023). Reliability and validity of the Chinese version of the anticipatory grief scale in caregivers of young and middle-aged patients with severe stroke. Journal of Hubei University of Medicine, 42, 676-680. doi: 10.13819/j.issn.2096-708X.2023.06.020

Marwit, S. J., and Meuser, T. M. (2002). Development and Initial Validation of an Inventory to Assess Grief in Caregivers of Persons With Alzheimer’s Disease. The Gerontologist 42, 751–765. doi: 10.1093/geront/42.6.751

Marwit, S. J., and Meuser, T. M. (2005). DEVELOPMENT OF A SHORT FORM INVENTORY TO ASSESS GRIEF IN CAREGIVERS OF DEMENTIA PATIENTS. Death Studies 29, 191–205. doi: 10.1080/07481180590916335

Meichsner, F., Schinköthe, D., and Wilz, G. (2016). The Caregiver Grief Scale: Development, Exploratory and Confirmatory Factor Analysis, and Validation. Clinical Gerontologist 39, 342–361. doi: 10.1080/07317115.2015.1121947

Mystakidou, K., Tsilika, E., Parpa, E., Katsouda, E., Sakkas, P., and Soldatos, C. (2005). Life before death: identifying preparatory grief through the development of a new measurement in advanced cancer patients (PGAC). Support Care Cancer 13, 834–841. doi: 10.1007/s00520-005-0797-4

Önal, G., Keser, E., and GÜN, Z. T. (2023). Validity and Reliability Study of the Prolonged Grief Disorder- Caregiver Turkish Form. Turkish Journal of Psychiatry 35, 46–55. doi: 10.5080/u27035

Periyakoil, V. S., Kraemer, H. C., Noda, A., Moos, R., Hallenbeck, J., Webster, M., et al. (2005). The development and initial validation of the Terminally Ill Grief or Depression Scale (TIGDS). Int. J. Methods Psychiatr. Res. 14, 203–212. doi: 10.1002/mpr.8

Theut, S. K., Jordan, L., Ross, L. A., and Deutsch, S. I. (1991). Caregiver’s Anticipatory Grief in Dementia: A Pilot Study. Int J Aging Hum Dev 33, 113–118. doi: 10.2190/4KYG-J2E1-5KEM-LEBA

Xin, D. J. (2017). Study on grief of advanced cancer patients and their families (Master’s thesis, Southwest Medical University, Luzhou, China)
